# Probing Cell Redox State and Glutathione-Modulating Factors Using a Monochlorobimane-Based Microplate Assay

**DOI:** 10.3390/antiox11020391

**Published:** 2022-02-15

**Authors:** Rezeda A. Ishkaeva, Mohamed Zoughaib, Alexander V. Laikov, Plamena R. Angelova, Timur I. Abdullin

**Affiliations:** 1 Department of Biochemistry, Biotechnology and Pharmacology, Institute of Fundamental Medicine and Biology, Kazan Federal University, 18 Kremlyovskaya St., 420008 Kazan, Russia; rezaahmadishina@kpfu.ru (R.A.I.); zmokhamed@kpfu.ru (M.Z.); avlajkov@kpfu.ru (A.V.L.); 2 Department of Clinical and Movement Neurosciences, Queen Square Institute of Neurology, University College London, Queen Square, London WC1N 3BG, UK; p.stroh@ucl.ac.uk

**Keywords:** glutathione status, oxidative status, mammalian cells, monochlorobimane, microplate assay, redox-modulating factors, LC–MS/MS

## Abstract

Thiol compounds including predominantly glutathione (GSH) are key components of redox homeostasis, which are involved in the protection and regulation of mammalian cells. The assessment of cell redox status by means of in situ analysis of GSH in living cells is often preferable over established assays in cell lysates due to fluctuations of the GSH pool. For this purpose, we propose a microplate assay with monochlorobimane (MCB) as an available fluorescent probe for GSH, although poorly detected in the microplate format. In addition to the new procedure for improved MCB-assisted GSH detection in plate-grown cells and its verification with GSH modulators, this study provides a useful methodology for the evaluation of cell redox status probed through relative GSH content and responsiveness to both supplemented thiols and variation in oxygen pressure. The roles of extracellular interactions of thiols and natural variability of cellular glutathione on the assay performance were emphasized and discussed. The results are of broad interest in cell biology research and should be particularly useful for the characterization of pathological cells with decreased GSH status and increased oxidative status as well as redox-modulating factors.

## 1. Introduction

Thiol-containing biomolecules are a key component of protecting antioxidant and regulatory systems in mammalian cells. The tripeptide glutathione (L-γ-glutamyl-L-cysteinyl-glycine) is the predominant thiol with an intracellular concentration greatly exceeding that of sulfur amino acids, including the immediate precursor L-cysteine [1,2]. It has multiple activities that rely on the reactions of cysteine thiol catalyzed by a series of glutathione-dependent enzymes. These reactions involve the elimination of reactive oxygen and nitrogen species (ROS and RNS), inactivation of harmful electrophilic compounds and α-oxoaldehydes, as well as redox signaling in cells [3].

Intracellular levels of glutathione in reduced form (GSH) and oxidized form (GSSG), as well as the GSH/GSSG ratio, are crucial parameters of redox homeostasis [4], which regulate the transition between main cellular processes including proliferation, differentiation, senescence and programmed death [5]. Regulatory mechanisms of the GSH/GSSG couple are based on the direct suppression of ROS/RNS-mediated signaling and modulation of the activity of redox-sensitive proteins via thiol–disulfide conversions of cysteine residues including the formation of mixed cysteine-glutathione disulfide. The depletion of GSH due to an abnormal production of ROS/RNS and impaired antiradical defense underlies a series of pathological conditions such as excessive apoptosis, degeneration and chronic inflammation [6], as well as tumor transformation [7] and viral propagation [8].

Therefore, effective assessment of the redox status of mammalian cells viewed through glutathione content is of paramount importance for fundamental research of cell functioning and pathogenesis, as well as the discovery of antiradical and redox-modulating drugs capable of cell protection and functional regulation. Various methods for the detection of glutathione in vitro and in vivo, particularly based on optical, electrochemical and nuclear magnetic resonance spectroscopy techniques, have been proposed [9,10]. Nevertheless, there is still a need for rapid, robust and high-throughput assays for GSH quantification and assessment of cell redox status.

The assays based on gas/liquid chromatography–mass spectrometric detection [11,12,13] or glutathione reductase-assisted optical detection [14,15] are established ones for the analysis of both GSH and GSSG. The above techniques, however, require cell lysis accompanied by difficult-to-control autooxidation of the GSH pool, hardly consider rapid redox fluctuations and are also laborious and/or sufficiently expensive for high-throughput tasks. The luminescence probes provide an important, often preferable, tool for in situ detection of GSH in whole cells, taking into consideration intrinsic fluctuation and compartmentalization of the tripeptide (see the review [16]).

In spite of the unflagging interest in the development of new luminescence probes for GSH, such as bis-pyrene-Cu(II) complex [17], vinyl-functionalized boron-dipyrromethene (4,4-difluoro-4-bora-3a,4a-diaza-s-indacene) (BODIPY) [18,19] and sulfinyl naphthalimides [20], monochlorobimane (MCB) remains one of the most selective and available fluorescent dyes for this purpose. It has been widely used to assess physiological variations of GSH in different types of mammalian cells, mainly using sensitive fluorescence microscopy techniques [21,22,23,24,25]. The corresponding microplate analysis is complicated by poor detection of MCB fluorescence in a relatively short-wavelength range. The reported microplate assay with MCB deals with the detection of GSH depletion by cytotoxic drugs [26] and does not address the analytical possibilities for the assessment of redox status as well as recovery of GSH in the cells. Such an assessment is complicated compared to the analysis of GSH depletion due to cellular mechanisms that control upper GSH level as well as significant natural fluctuations of both GSH and GSSG, which have not been addressed often in the literature.

In this work, a substantially improved MCB-based microplate assay was elaborated to provide convenient and multitasking analysis of GSH in mammalian cells. The assay was for the first time employed to characterize different redox states in the cells viewed through GSH content, sensitivity toward exogenous thiols and ambient oxygen concentration in relation to ROS levels. Practical details concerning the analysis of GSH-modulating factors were considered, focusing on GSH replenishment and depending on cell redox status. The results justify the proposed assay as a useful tool for cell biology studies and the screening of redox-modulating factors.

## 2. Materials and Methods

### 2.1. Materials

Reduced glutathione, oxidized glutathione (98%) and 1,4-naphthoquinone (menadione, 97%) were purchased from Acros Organics. Monochlorobimane and MitoSOX Red Mitochondrial Superoxide Indicator were purchased from Thermo Fisher Scientific. 2′,7′-dichlorofluorescin diacetate (DCFDA) and L-buthionine-sulfoximine (BSO) were purchased from Sigma-Aldrich. Milli-Q grade water (Milli-Q^®^ Advantage A10, Merck Millipore, Darmstadt, Germany) was used to prepare buffers and solutions. HPLC-grade acetonitrile was purchased from Merck. Cell culture media and reagents were purchased from Paneco (Moscow, Russia).

### 2.2. Cell Culture

Human glioma-derived cell lines LN-229 and SNB-19 (ATCC) were used. The cells were cultured aseptically in DMEM supplemented with 10% fetal bovine serum (FBS), 2 mM L-glutamine, 100 U/mL penicillin and 100 µg/mL streptomycin at 37 °C in humidified air atmosphere with 5% CO_2_. Swiss mouse embryo fibroblast (NIH 3T3) cell lines (ATCC) were used. 3T3 cells were cultured under the same conditions but in α-MEM with 10% FBS.

### 2.3. Conditions for MCB Analysis

A 100 mM stock MCB solution in DMSO was used. Hank’s balanced salt solution (HBSS) additionally supplemented with 10 mM HEPES (pH = 7.4) was used to prepare working solutions. Final concentrations of the compounds were in the range 1–25 μM (MCB) and 0.01–10 mM (glutathione).

MCB fluorescence signals were recorded at an excitation wavelength of 380 nm on a spectrofluorometer FL3-221-NIR (Horiba Jobin Yvon, Kyoto, Japan) using a 1 cm quartz cuvette (slit 3 nm) and on an Infinite 200 PRO microplate analyzer (Tecan, Männedorf, Switzerland) using 96-well cell culture polystyrene plate (SPL Life Sciences, Pocheon, Korea). The MCB signal in adherent cells was detected using the parameters as follows: ‘bottom’ mode, emission wavelength 480 nm, gain 100, integration time 20 µs, number of flashes 25.

### 2.4. HPLC Analysis of GSH and Its Adducts

Conjugation of GSH with menadione and MCB was detected by HPLC method using a Dionex UltiMate 3000 HPLC system (Thermo Scientific, Waltham, MA, USA) and a reverse-phase Kromasil C18 column. Briefly, GSH at different concentrations (0.01, 0.1, 1 mM) was incubated with MCB (10 μM) or menadione (200 μM) in HBSS for 1 and 2 h, respectively. Gradient elution with water/acetonitrile mobile phase was applied at a constant flow rate of 0.5 mL/min. Eluted menadione and MCB (both free and conjugated) were analyzed by UV-diode (λ = 260 nm) and fluorescent (λ = 482 nm) detectors, respectively.

To quantify intracellular GSH content, the cells were grown in 6-well plate for 24 h, then harvested by trypsinization and counted. The cells (1 × 10^6^) were subsequently collected by centrifugation, lysed in 100 µL of 1% trifluoroacetic acid (TFA) and frozen at −80 °C, followed by thawing and 10-fold dilution with milli-Q water (to a final volume of 1 mL and a TFA concentration of 0.1%). The samples were additionally sonicated on ice for 3 min and centrifuged at 13,000 × *g* for 12 min at 4 °C. The supernatants were subjected to reverse-phase HPLC analysis in isocratic mode with a flow rate of 0.5 mL/min at a detection wavelength of 215 nm. The eluent composition consisted of water/acetonitrile (95:5, *v*/*v*), 0.1% TFA and 12 mg/mL of sodium perchlorate NaClO_4_ [27]. The linear calibration graphs were obtained by plotting GSH concentration (1–250 µM) against the detected peak area, which showed a linear relationship (*r^2^* = 0.9911).

### 2.5. Microplate GSH Detection in Intact and Treated Cells

The cells were seeded in a 96-well plate at a density of 2 × 10^4^ cells per well in the culture medium and grown overnight. The medium was replaced by HBSS prior to adding the compounds. The cells were exposed to menadione (25, 50, 100 µM), glutathione (0.01, 0.1, 1, 10 mM) and MCB (5 µM) in HBSS in CO_2_ incubator. The compounds were added to the cells in different combinations/treatment orders, as detailed in the Section 3.2. Briefly, upon co-treatment, menadione was initially added for 1 h followed by addition of GSH (10×) for 1 h (without removing menadione); staining with MCB was performed in the presence of modulators. Upon sequential treatment, the cells were sequentially exposed to pure solutions of menadione, GSH and MCB (each compound was added for 1 h after removal of previous one).

In addition, the cells were cultured in the presence of BSO (0.4–200 µM) in the culture medium for 24 h followed by the exposure to GSH and/or MCB for 1 h in HBSS. To assess the mechanism of GSH internalization, the cells were exposed to GSH in HBSS with adjusted pH values (7.4 and 5.0) and in the presence of active transport inhibitors cocktail (0.065 mg/mL NaN_3_, 0.1 mg/mL NaF).

As a measure of intracellular GSH level, the relative increment of MCB fluorescence between 60 min (I_60_) and 0 min (I_0_) of cell staining with the probe was calculated in each well separately (*n* = 6) using the formula: (I_60_-I_0_)/I_0_.

To assess the effect of oxygen tension, the cells were cultured for 5 h in Bactrox Hypoxia Chamber (Shel Lab, Cornelius, OR, USA) at both O_2_ and CO_2_ concentrations of 5% and temperature of 37 °C. Any exposure of the cells to ambient atmosphere during staining and analysis with MCB was avoided.

### 2.6. ROS Assessment

LN-229 and SNB-19 cells were collected by trypsinization and washed with and suspended in HBSS at a density of 10^6^ cells/mL. The cells were stained with 5 µM MitoSOX or 20 µM DCFDA for 20 min at 37 °C to analyze mitochondrial and cytoplasmic ROS, respectively. The analysis was performed on a Guava EasyCyte 8HT flow cytometer (Millipore, Darmstadt, Germany).

### 2.7. LC–MS/MS Analysis of GSH/GSSG Ratio

The cells were seeded in 6-well plates (5 × 10^5^ cells per well), cultured for 24 h and then harvested as follows. The cells were trypsinized, pelleted and lysed with 1% TFA solution or directly scraped with 1% TFA solution; both extractions were conducted on ice for 10 min. The cell lysates were frozen at −80 °C, thawed and diluted with mQ water to obtain a final cell density of ca. 1 × 10^6^ cells/mL and TFA concentration of 0.1%. GSH and GSSG standards in TFA solutions were used.

Liquid chromatography–tandem mass spectrometry (LC–MS/MS) analysis in multiple reaction monitoring (MRM) mode was performed to quantify GSH and GSSG in the cells. The analysis was performed using an Infinity 1290 HPLC system (Agilent, Santa Clara, CA, USA) combined with a QTRAP 6500 triple quadrupole mass spectrometer (ABSciex, Singapore) equipped with an electrospray ionization source (ESI). Reverse-phase chromatographic separation of the samples was conducted using a Discovery HS C18 column (3 μm, 5 cm × 2.1 mm, Supelco, Bellefonte, PA, USA) with mobile phases consisting of A (99.9% water, 0.1% formic acid) and B (94.9% acetonitrile, 5% water, 0.1% formic acid). Elution mode was as follows (phase B, %): 0–0.5 min—0%, 0.5–2.5 min—0→50%, 2.5–2.6 min—50→99%, 2.6–3.6 min—99%, 3.6–3.8 min—99→0%, 3.8–5.0 min—0%. The flow rate was 0.4 mL/min, and the temperature was 40 °C. Negatively charged ions were generated using a Turbo Spray Ion Drive as an ESI with the optimized parameter settings as follows: spray voltage 4500 V, nebulizer gas pressure 60 psi, auxiliary gas pressure 60 psi, curtain gas pressure 35 psi, temperature 500 °C. The quantifier/qualifier ions *m*/*z*, declustering potential and collision energy were optimized using an automated ‘Compound optimization’ algorithm of the Analyst 1.6.2 software (ABSciex). The MRM transitions were: *m*/*z* 305.9→272.0, *m*/*z* 305.9→143.0 (GSH), *m*/*z* 611.0→306.0 and *m*/*z* 611.0→271.9 (GSSG). The analytes were quantified according to the peak area of corresponding MRM transition using a MultiQuant 3.0.2 software (AB Sciex). LC–MS-grade solutions were used for the analysis. 

### 2.8. Statistical Analysis

The data were presented as mean ± standard error (*n* = 6), unless otherwise indicated. Statistical significance was determined by one-way analysis of variance (ANOVA) followed by Tukey’s multiple comparison post-test (* *p* < 0.05, ** *p* < 0.01, *** *p* < 0.001).

## 3. Results

### 3.1. Establishment of Microplate Assay

**Figure 1:**
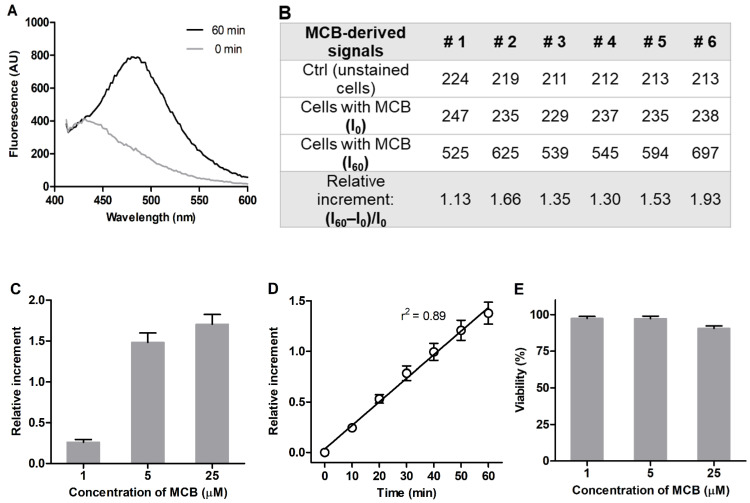
Optimization of MCB detection in 3T3 fibroblasts grown in 96-well plates. (**A**) Fluorescence emission spectra of 5 μM MCB in adhered 3T3 fibroblasts in HBSS (λ_ex_ = 380 nm, ‘bottom’ mode). (**B**) Representative fluorescence values for MCB-stained cells (AU) collected from six wells. (**C**) Concentration dependence of MCB signal (60 min incubation). (**D**) Time dependence of MCB signal (5 μM MCB). (**E**) Viability of MCB-treated cells according to MTT assay (relative to viability of untreated cells = 100%). Mean values ± SEM (*n* = 6) are shown.

3T3 mouse embryonic fibroblasts were used as model mammalian cells sensitive to redox-modulating factors [28,29,30]. The cells were grown in polystyrene 96-well microplates and maintained as a subconfluent monolayer to achieve sufficient cell density while avoiding contact-induced cell quiescence. The MCB fluorescence was acquired from the stained cells in HBSS at λ_em_ = 486 nm (Figure 1A) without any shift in the emission wavelength compared to quartz cuvette. The intracellular signal of GSH was measured as a relative increment of the MCB fluorescence between 60 and 0 min of incubation separately in each of the six wells ((I_60_–I_0_)/I_0_) (Figure 1B). The proposed procedure allowed us to account for signal variability related to background fluorescence of the plate material, extracellular MCB as well as for some variation in the density of plated cells, providing accurate and more consistent detection of MCB fluorescence in the cells. Saturation of the signal was observed at an MCB concentration of 5 μM, which was chosen to perform the assay (Figure 1C). The duration of cell staining with MCB was confined to 60 min, which ensured a linear increase of the signal (Figure 1D) and was not accompanied by a decrease in cell viability (Figure 1E).

### 3.2. Assay Verification Using GSH Modulators

#### 3.2.1. Co-Treatment Conditions

**Figure 2:**
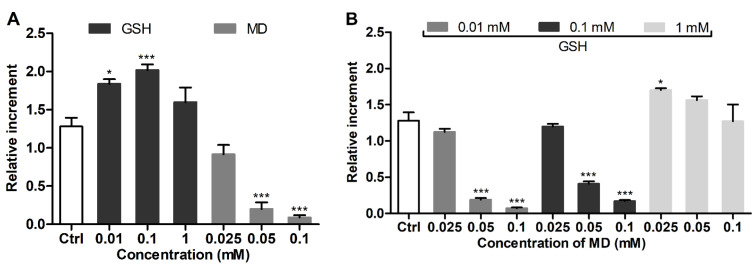
Effect of (**A**) supplemented GSH or menadione (MD) and (**B**) their mixture on MCB signal in 3T3 fibroblasts. The cells were treated with the modulators for 1 h and stained with MCB for additional 1 h (in the presence of modulators). For co-treatment, the cells were pre-exposed to MD followed by addition of GSH. Mean ± SEM (*n* = 6, * p < 0.05, *** p < 0.001 vs. Ctrl) are shown; Ctrl is the signal of MCB alone.

3T3 fibroblasts were treated with GSH as a modulator of cellular GSH and menadione as a GSH depletor [31] separately (Figure 2A) and in the mixture (Figure 2B), followed by the addition of MCB. The cells were exposed to MCB without removing the modulating compounds from the extracellular solution in order to assess their equilibrium effect on the GSH level. MCB fluorescence was noticeably increased by supplemented GSH at a concentration as low as 0.01 mM (Figure 2A). Menadione (0.025–0.1 mM) induced a concentration-dependent decrease of the signal (Figure 2A); the effect of menadione was prevented by co-supplemented GSH (0.01–1 mM) (Figure 2B). The reproducibility of the detected changes of the MCB signal in the treated cells was confirmed in independent experiments (Figure S1).

HPLC analysis showed that co-incubation of menadione and GSH in HBSS was accompanied by their conjugation (due to the formation of S-conjugate [32]) in proportion to the concentration of GSH (0.01–1 mM). This reveals the extracellular binding of menadione by GSH, which should affect the intracellular GSH-depleting effect under co-treatment conditions, although being a relevant cell protective mechanism of supplemented thiols against GSH depletors [33]. The conjugation of MCB with GSH also occurred in the solution, though requiring the presence of excess thiols (1 mM) (Figure S2). Nevertheless, even at lower concentrations, GSH increased MCB fluorescence per se (Figures S2 and S3), indicating that the probe can be activated non-enzymatically besides the adduct formation catalyzed by glutathione-S-transferase isoenzymes [22]. To assess the effect of extracellular interactions on the MCB-assisted detection of cellular GSH, the analysis was further performed upon a sequential addition of the compounds to the cells.

#### 3.2.2. Sequential Treatment Conditions

**Figure 3:**
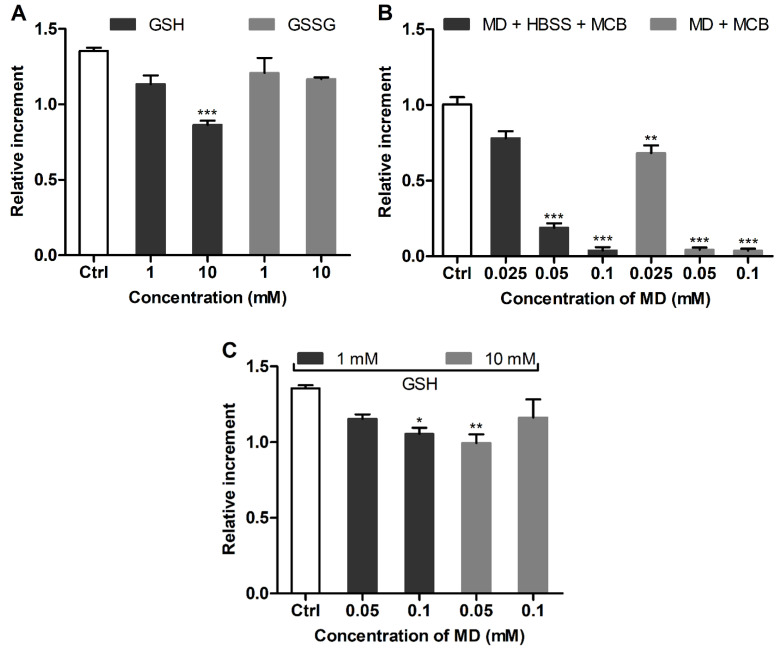
MCB signals in 3T3 fibroblasts under sequential 1 h treatment with GSH modulators. (**A**) Sequential exposure to GSH or GSSG and MCB. (**B**) Sequential exposure to menadione, plain HBSS (optionally) and MCB. (**C**) Sequential exposure to menadione, GSH and MCB. Mean ± SEM (*n* = 6, * p < 0.05, ** p < 0.01, *** p < 0.001 vs. Ctrl) are shown.

3T3 fibroblasts were treated sequentially to avoid extracellular GSH–MCB interactions, which likely occur at increased GSH concentrations. Sequential exposure of the cells to GSH and MCB (after removing GSH) was not accompanied by an increase in cellular GSH in a wide concentration range of supplemented GSH. This suggests that the aforementioned increase of MCB signal upon cell co-treatment with GSH (0.01/0.1 mM) and the probe (Figure 2A) is supported by transient equilibrium between extra- and intracellular GSH. Furthermore, sequential cell treatment with 1 and 10 mM GSH resulted in a noticeable decrease in cellular GSH, presumably due to some redox imbalance in the cells in the presence of excessive GSH concentrations (Figure 3A).

To account for menadione–GSH extracellular binding, the cells were sequentially exposed to pure solutions of menadione, GSH and MCB (each for 1 h). Control sequential treatment with menadione and MCB or with menadione, blank HBSS (1 h) and MCB provided comparable MCB signals (Figure 3B), suggesting that the GSH-depleting effect of menadione persists at least for 1 h after its withdrawal. Therefore, a compound of interest can be applied in this time interval (instead of blank HBSS) to reveal its GSH-restoring ability. Under these conditions, 1 and 10 mM GSH effectively replenished cellular GSH after depletion with menadione (Figure 3C).

**Figure 4:**
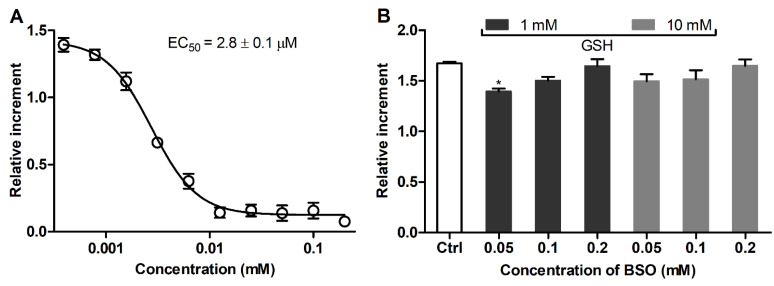
(**A**) Concentration-dependent GSH depletion by BSO in cultured 3T3 fibroblasts (24 h). (**B**) Restored GSH levels in 3T3 fibroblasts sequentially exposed to BSO (24 h) and GSH (1 h) according to MCB fluorescence. Mean ± SEM (*n* = 6, * p < 0.05 vs. Ctrl) are shown.

In addition, the effect of BSO as a specific inhibitor of GSH biosynthesis [25,34] was studied. BSO induced a well-defined dose-dependent decrease of GSH in the cultured cells with a half-maximal effective concentration (EC_50_) of 2.8 ± 0.1 μM (Figure 4A, 24 h). The GSH level in the BSO-treated cells was restored by post-treatment with 1 and 10 mM GSH (Figure 4B), further confirming that supplemented GSH allows for the replenishment of specifically depleted GSH pools in 3T3 fibroblasts.

### 3.3. Assessment of GSH Status in Glioblastoma Cells

**Figure 5:**
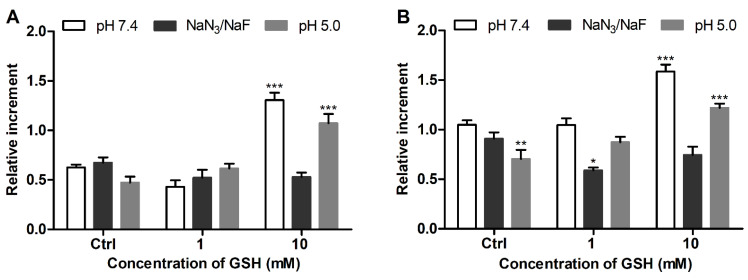
MCB signals in intact and GSH-treated glioblastoma cells (**A**) LN-229 and (**B**) SNB-19 under different conditions. Mean ± SEM (*n* = 6, * p < 0.05, ** p < 0.01, *** p < 0.001 vs. Ctrl) are shown.

Human glioblastoma cell lines LN-229 and SNB-19 were additionally assessed using the proposed assay. The cells were characterized by different GSH levels (i.e., LN-229 < SNB-19), both lower than in 3T3 fibroblasts, as revealed by the MCB fluorescence increment for the intact cells (Figure 5, Ctrl).

**Figure 6:**
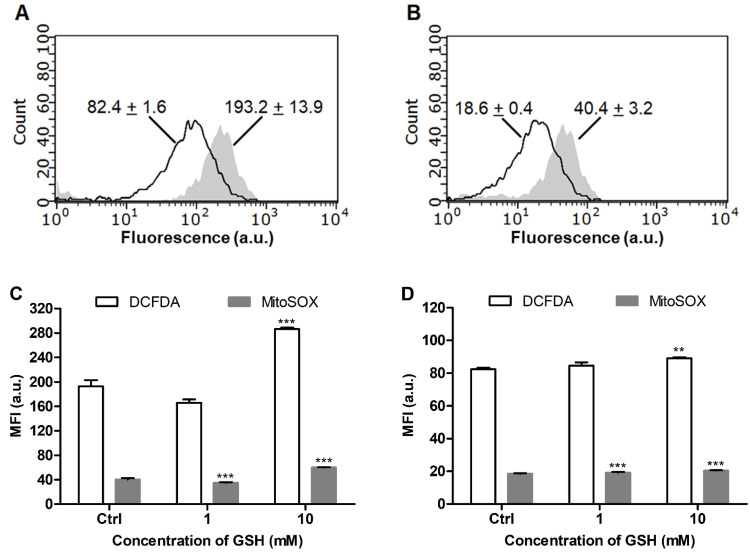
Distribution of (**A**) DCFDA and (**B**) MitoSOX fluorescence in intact LN-229 (filled curve) and SNB-19 (unfilled curve) cells according to flow cytometry (mean channel fluorescence for each curve is indicated). Mean signals of the probes after 1 h treatment of (**C**) LN-229 and (**D**) SNB-19 cells with GSH. Mean ± SEM (*n* = 3, ** p < 0.01, *** p < 0.001 vs. Ctrl) are shown.

The GSH status in the glioblastoma cells was in accordance with cytoplasmic and mitochondrial ROS levels, which were substantially increased in LN-229 cells (Figure 6A,B). LN-229 cells exhibited a much higher sensitivity toward supplemented GSH (10 mM), which induced drastic elevation of cellular GSH by a factor of 2.6 (Figure 5A), and this effect was accompanied by some increase in the ROS level (Figure 6C). The GSH-treated SNB-19 cells showed a lower increase of the GSH level (ca. 1.5-fold, Figure 5B) and a lack of significant ROS overproduction (Figure 6D).

Some acidification of the extracellular solution did not enhance the effect of supplemented GSH on the glioblastoma cells as could be expected in the case of some passive diffusion of the anionic tripeptide across the cell plasma membrane. Abolishment of this effect by NaN_3_/NaF (Figure 5) was observed, providing evidence for the involvement of an energy-dependent transport process in the intracellular penetration of GSH (or its metabolites).

### 3.4. Effect of Oxygen Concentration

**Figure 7:**
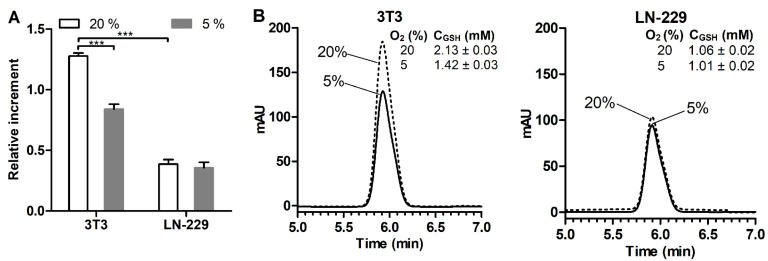
(**A**) MCB signals in intact 3T3 fibroblasts and LN-229 glioblastoma cells cultured under 20% and 5% oxygen levels for 5 h. (**B**) Corresponding reverse-phase HPLC chromatogramms of GSH in cell lysates (1 × 10^6^ cells per 1 mL) and found GSH concentrations. Mean ± SEM (*n* = 6, *** p < 0.001) are shown.

The assay performance was additionally verified using a hypoxic chamber, considering an important role of O_2_ partial pressure in the cell redox state [35,36,37]. 3T3 fibroblasts and LN-229 cells with greatly different GSH content were simultaneously cultured in a CO_2_ incubator (20% O_2_) or hypoxic chamber (5% O_2_) for 5 h, followed by the detection of GSH. The measurements were aimed at evaluating the effect of a short-term decrease in O_2_ concentration rather than modeling living tissue conditions. Under 5% O_2_, 3T3 fibroblasts were characterized by a more than 30% decrease in cellular GSH (compared to that for 20% O_2_), whereas the GSH level was maintained in LN-229 cells (Figure 7A). Parallel HPLC analysis showed a similar effect of O_2_ pressure reduction on the cellular GSH to that observed by the MCB assay (Figure 7B vs. Figure 7A). Furthermore, the HPLC data confirmed a markedly increased GSH level in 3T3 cells over LN-229 cells, although there was lower difference in the GSH level between these two cell lines. These results (Figure 7) can be explained by a potential prooxidant impact of the 20% O_2_ level (compared to the 5% O_2_ level found in some normal tissues [38]), causing ROS overproduction in responsive mammalian cells [35,36,37,39]. Therefore, attenuation of such a prooxidant O_2_ tension could expectedly alter the GSH–ROS equilibrium, leading to a compensatory decrease of GSH biosynthesis in 3T3 cells. The increased oxidative status of LN-229 cells should render them less sensitive to O_2_ change (Figure 7).

## 4. Discussion

Few studies assess cellular GSH using the microplate method with the MCB probe [26,40,41], despite its importance for fundamental, toxicological and pharmacological research [7,8,42]. This is due to a decreased sensitivity of the detection of MCB fluorescence in the cells grown on polystyrene plates, thus requiring optimization of the analysis for a particular task. The MCB signal optimized here in a subconfluent cell monolayer was only 2–2.5 times higher than the background signal (Figure 1A). Such a signal increment observed for 3T3 fibroblasts with an increased GSH level seems to be the maximal for the microplate format assay.

To the best of our knowledge, there is a single report on the optimization of an MCB-based microplate assay for GSH-depleting compounds [26]. It proposes to assess the change of MCB fluorescence per 1 min recorded during a 10 min detection period as an analytical signal. In our study, the relative increment of the MCB signal (I_60_–I_0_)/I_0_ (Figure 1B) was measured as a more sensitive parameter. The measurements were performed separately in each well for an extended number of wells (*n* = 6) to account for well-to-well variability and increase reproducibility of the analysis.

According to the optimized assay conditions, 5 μM of MCB was sufficient to saturate the plated cells during 60 min of incubation (Figure 1D). This MCB concentration is one order lower than the previously reported ones (50 μM [25], 40 μM [26]) and should be preferable in terms of selectivity. It also ensures extended linear dependence of the MCB signal on cell staining time up to 60 min, which should better account for signal variations due to fluctuations in temperature and gas composition, among other conditions upon cell manipulations.

The proposed assay revealed a clear difference in the GSH level in mammalian cells studied, i.e., 3T3 > SNB-19 > LN-229 cells. To further characterize the redox state of the cells, their responsiveness toward supplemented GSH was assessed as an established GSH-replenishing compound [43,44,45]. In spite of its relatively poor cellular uptake compared to GSH esters [28], the added GSH at millimolar concentrations was effective in changing cellular GSH. The effect of supplemented GSH was energy-dependent (Figure 5) in accordance with the established mechanism of GSH uptake as a result of extracellular degradation by γ-glutamyl transpeptidase and dipeptidases into amino acids carried by membrane transporters [42,45]. Our data suggest that supplemented GSH preferably increases the GSH level in cells with an originally decreased GSH status. In ‘GSH-rich’ 3T3 fibroblasts, added GSH was even capable of decreasing this level (Figure 3A), presumably in association with reduction stress under excessive GSH involving oxygen-dependent overproduction of ROS [42]. Notably, these cells were rendered sensitive to GSH replenishment after the specific inhibition of γ-glutamylcysteine synthetase by BSO (Figure 4) or treatment with menadione (Figure 2 and Figure 3), which depletes GSH both via S-conjugation and superoxide radical production [31,46,47]. Thus, the treatment of 3T3 fibroblasts with menadione (1 h in HBSS) or BSO (24 h in culture medium) in situ generates the cells with impaired GSH status, which could be used to assess GSH-replenishing compounds.

Furthermore, using human glioblastoma cells, it was shown that supplemented GSH is capable of increasing cellular GSH in proportion to the oxidative status of the cells (LN-229 > SNB-19). The latter status can be assessed through the detected GSH level (Figure 5) and confirmed by cytoplasmic and mitochondrial ROS production (Figure 6). These data further support the usefulness of the proposed assay for the characterization of the redox state of mammalian cells, which should determine their sensitivity to GSH-modulating factors. In addition, the results show that profound elevation of cellular GSH may cause ROS overproduction, as observed for LN-229 cells (Figure 6C) attributed to some unbalanced GSH pools, and these also demonstrate the double-faced nature of antioxidants.

The role of the O_2_ level as a modulating factor for cellular GSH was emphasized using the assay. In particular, ‘GSH-rich’ 3T3 fibroblasts responded to a brief decrease of O_2_ pressure from 20% to 5% by lowering cellular GSH, whereas ‘GSH-poor’ LN-229 cells remained unresponsive (Figure 7). These results are explained by the observations that 20% O_2_ pressure (often referred to as a ‘normoxic’ [38]) may mediate ROS overproduction in mammalian cells [36,38,43,48]. As previously shown, skin fibroblasts [35,48] and mesenchymal stem cells [36] cultured under 20% O_2_ feature an increased oxidative status (over the cells cultured under ≤5% O_2_), strongly affecting proliferation, differentiation, senescence phenotype and metabolic activity of the cells. Therefore, attenuation of O_2_ tension in 3T3 fibroblasts pre-adapted to 20% O_2_ is expected to induce some ROS inhibition along with a compensatory decrease in GSH as a major redox buffer in accordance with the previous data for HSF [48]. Raised oxidative status of LN-229 cells seemingly renders them weakly sensitive to O_2_ concentration in contrast to 3T3 cells (Figure 7).

Our data also show that the assay is readily applicable to be performed in a hypoxia chamber and prove the O_2_ level as a potential factor affecting the assay performance. Interestingly, according to the annual dynamics in the area, partial density of atmospheric O_2_ noticeably decreases during the transition from winter to summer months (Figure S4), similarly to the meteorological data for other cities [49]. Repetitive analysis of the cells showed that the GSH level in 3T3 fibroblasts but not in LN-229 cells was also significantly lowered in spring–summer compared to the winter period (data not shown). Based on the above observations, this phenomenon could be associated with a seasonal change of O_2_ pressure (probably in association with other factors).

Altogether, this study for the first time proposes an elaborated MCB-based microplate assay for the assessment of redox status in mammalian cells probed through initial GSH levels and their changes in response to exogenic GSH and O_2_ pressure. The proposed assay should be of wide interest in physiological and pharmacological in vitro studies, and it particularly allows the identification of cell lines/cultures with decreased GSH status, potentially sensitive to GSH-modulating factors, as cellular models of degenerative diseases [50,51,52]. We believe that the assay can be regarded as a useful tool to supplement other methods for glutathione analysis in the cells.

This discussion additionally emphasizes the role of significant variability of cellular GSH resulting from natural metabolic fluctuations and/or assay conditions. The GSH level in 3T3 fibroblasts was earlier shown to be associated with proliferative rate, cell cycle parameters and telomerase activity, and it was noticeably altered upon extended cell culture [30,53]. In particular, up to a fourfold change in this level was reported upon active growth of 3T3 cells in culture [53]. Furthermore, the existing studies also provide controversial GSH/GSSG ratios in the cultured cells. Table S1 summarizes corresponding GSH/GSSG values for different fibroblast cells. Notably, for 3T3 cells, the reported GSH/GSSG ratio varies from ca. 10 to 400 (Table S1). Taken into account that the overall GSH/GSSG ratio in mammalian cells is usually ≥100 [54], much lower values should result from inaccuracies in glutathione quantification. This especially concerns GSSG detection, since GSH autooxidation in the cell lysate may result in a considerable overestimation of GSSG, leading to a decreased GSH/GSSG ratio. To overcome this overestimation, GSH can be quenched by thiol-alkylating agents [55,56,57]. Effective analysis of underivatized GSH/GSSG in cell samples under acidic conditions was also proposed [58].

The reported HPLC or HPLC–MS-based protocols for simultaneous analysis of GSH and GSSG in cell lysates generally provide increased ratios, namely comparable to or over 100. According to these analyses, the estimated GSH/GSSG ratio in lymphocytes from 20 healthy individuals varied from 42 to 432 [57], whereas for 16 human cell lines/cultures it was in the range from 156 to 868 [55]. Among the latter cell library, the cells with increased GSH level generally were not characterized by an increased GSH/GSSG ratio, and no clear relationships between the ratio and normal-to-cancerous phenotypes could be revealed from these data [55]. Moreover importantly, during 24 h culture, the GSH/GSSG ratio in 3T3-L1 cells showed a physiological sinusoid-like fluctuation with a difference of ca. 2.6 times between the lowest (150) and the highest (400) values [59]. Together, these data suggest that the characterization of cell redox status via GSH/GSSG values per se might be complicated. This further supports the importance of microplate methods to probe this status in situ as well as careful maintenance of the conditions for cell growth, which are often not properly addressed.

Nevertheless, additional HPLC–MS/MS analysis was applied to assess the GSH/GSSG ratio in 3T3 and glioblastoma cells under analogous conditions to those used for the MCB-assisted assay. The analysis is based on previous protocols, which allow for the inhibition of GSH autooxidation in TFA solutions [27,58]. Furthermore, the direct MRM detection of underivatized GSH and GSSG in negative ionization mode was optimized (Section 2.5, Figures S5–S7) to avoid partial reduction of GSSG in the ion source commonly observed for disulfide compounds in positive ionization mode [60].

According to the obtained data (Table 1), 3T3 fibroblasts exhibit relatively high GSH/GSSG ratios of 161 (pre-trypsinized cells) and 265 (scraped cells), which are within the range reported for continuous 24 h HPLC analysis of 3T3-L1 cells [59], also supporting the lack of significant GSH oxidation in the cell samples. LN-229 cells were characterized by significantly lower ratios compared to 3T3 cells, generally in accordance with decreased GSH status and increased oxidative status of the former cells revealed by the MCB-based assay. The results also show that additional cell trypsinization prior to lysis is accompanied by a considerable decrease of the GSH/GSSG ratio in both types of cells, presumably due to a disturbing effect on the plasma membrane and/or GSH leakage, further supporting the importance of using specified conditions for the analysis of cell redox parameters.

## 5. Conclusions

Despite being one of the most popular fluorescent probes for GSH, MCB is difficult to detect in the microplate format. A reliable in situ assessment of GSH modulation in living cells is of considerable interest in research related to redox homeostasis, GSH-replenishing and cytoprotective therapy. To achieve this goal, we have developed a substantially improved MCB-based assay for GSH, which takes into consideration significant fluctuations in GSH content in cultured mammalian cells and potential extracellular reactions of thiols. The assay allowed for the characterization of the GSH status/oxidative status of the cells according to their MCB signals and responsiveness to GSH-modulating factors. Important conditions to assess GSH replenishment in the cells were revealed using supplemented GSH as a model thiol and promising therapeutic agent [43]. The proposed assay should be of broad interest for studying cell redox parameters and GSH modulators, and it can be extended to be used with newly developing fluorescent probes for cellular thiols.

## Figures and Tables

**Table 1:** GSH/GSSG ratios in cell lysates (1 × 10^6^ cells in 0.1% TFA) according to HPLC–MS/MS analysis (Section 2.7). Mean ± SD are shown (*n* = 3, ** p < 0.01, *** p < 0.001 vs. LN-229 cells; ^###^ *p* < 0.001 vs. pre-trypsinized cells).

Samples	3T3 Fibroblasts	LN-229 Cells
Pre-trypsinized	161 ± 9 **	127 ± 4
Scraped	265 ± 14 ***^,###^	193 ± 7 ^###^

## Data Availability

The data presented in this study are available within the article and Supplementary Materials.

## Supplementary Materials

The following supporting information can be downloaded at: https://www.mdpi.com/article/10.3390/antiox11020391/s1, Table S1: Reported GSH/GSSG ratio values in different fibroblast cells; Figure S1: Effect of supplemented GSH or menadione and their mixture on MCB signal in 3T3 fibroblasts; Figure S2: Reverse-phase HPLC chromatograms of menadione and MCB incubated with GSH; Figure S3: Fluorescence emission spectra of MCB in the presence of GSH; Figure S4: Annual dynamics of partial density of oxygen in Kazan; Figure S5: Negative ion spectra generated in flow injection analysis of GSH and GSSG; Figure S6: Product ion spectra obtained from GSH and GSSG parent ions; Figure S7: Calibration graphs for GSH and GSSG.
