# Supplementary material for: Probing Cell Redox State and Glutathione-Modulating Factors Using a Monochlorobimane-Based Microplate Assay
Source: Antioxidants (Basel). 2022 Feb 15;11(2):391. doi: 10.3390/antiox11020391 (PMC8869332; doi:10.3390/antiox11020391)
Supplement: Supplementary file 1 [file antioxidants-11-00391-s001.zip › antioxidants-1577543-supplementary.pdf]

Probing Cell Redox State and Glutathione-Modulating Factors Using a Monochlorobimane-Based Microplate Assay

Rezeda A. Ishkaeva [1], Mohamed Zoughaib [1], Alexander V. Laikov [1], Plamena R. Angelova [2] and Timur I. Abdullin [1,*]

[1] Department of Biochemistry, Biotechnology and Pharmacology, Institute of Fundamental Medicine and Biology, Kazan Federal University, 18 Kremlyovskaya St., 420008 Kazan, Russia; rezaahmadishina@kpfu.ru (R.A.I.); zmokhamed@kpfu.ru (M.Z.); avlajkov@kpfu.ru (A.V.L.)
[2] Department of Clinical and Movement Neurosciences, Queen Square Institute of Neurology, University College London, Queen Square, London WC1N 3BG, UK; p.stroh@ucl.ac.uk
* Correspondence: timur.abdullin@kpfu.ru or tabdulli@gmail.com; Tel.: +7-843-236-7640

Table S1. Reported GSH/GSSG ratio values in fibroblast cells.

Cells	Method	GSH/GSSG	Reference
HSF (skin biopsy)	Spectrophotometric detection with DTNB amplified by glutathione reductase (GSH quencher is 2-vinylpiridine)	**~205**	DOI:10.1016/j.biocel.2017.05.005
NIH/3T3	HPLC analysis after derivatization with iodoacetic acid and 1-fluoro-2,4-dinitrobenzene	**10-19**	DOI:10.1016/S0167-4889(99)00142-1
NIH/3T3	Spectrophotometric detection with DTNB amplified by glutathione reductase (GSH quencher is 2-vinylpiridine)	**14.5**	DOI:10.1002/(SICI)1097-4652(199806)175:3<359::AID-JCP14>3.0.CO;2-0
3T3-L1 cells	HPLC analysis after derivatization with iodoacetic acid and 1-fluoro-2,4-dinitrobenzene	**~150 – 400**	DOI:10.1002/jcp.22542
HSF (foreskin)	Glutathione-S-transferase based assay for GSH; HPLC analysis for GSSG (GSH quencher is N-ethylmaleimide).	**124**	DOI:10.1111/j.0906-6705.2004.00112.x
HSF (ATCC)	Spectrophotometric detection with DTNB amplified by glutathione reductase (GSH quencher is 2-vinylpiridine)	**~41**	DOI:10.3390/antiox8090382
BALB/c3T3	HPLC analysis after derivatization with N-ethylmaleimide and bathophenanthroline disulfonic acid	**90**	DOI:10.1016/j.canlet.2003.11.007

Citation: Ishkaeva, R.A.; Zoughaib, M.; Laikov, A.V.; Angelova, P.R.; Abdullin, T.I. Probing of Cell Redox State and Glutathione Modulating Factors Using a Monochlorobimane-Based Microplate Assay. *Antioxidants* **2022**, *11*, 391. https://doi.org/10.3390/antiox11020391

Academic Editors: José Joaquín Cerón and Camila Peres Rubio

Received: 14 January 2022
Accepted: 11 February 2022
Published: 15 February 2022

Publisher's Note: MDPI stays neutral with regard to jurisdictional claims in published maps and institutional affiliations.

Copyright: © 2022 by the authors. Licensee MDPI, Basel, Switzerland. This article is an open access article distributed under the terms and conditions of the Creative Commons Attribution (CC BY) license (https://creativecommons.org/licenses/by/4.0/).

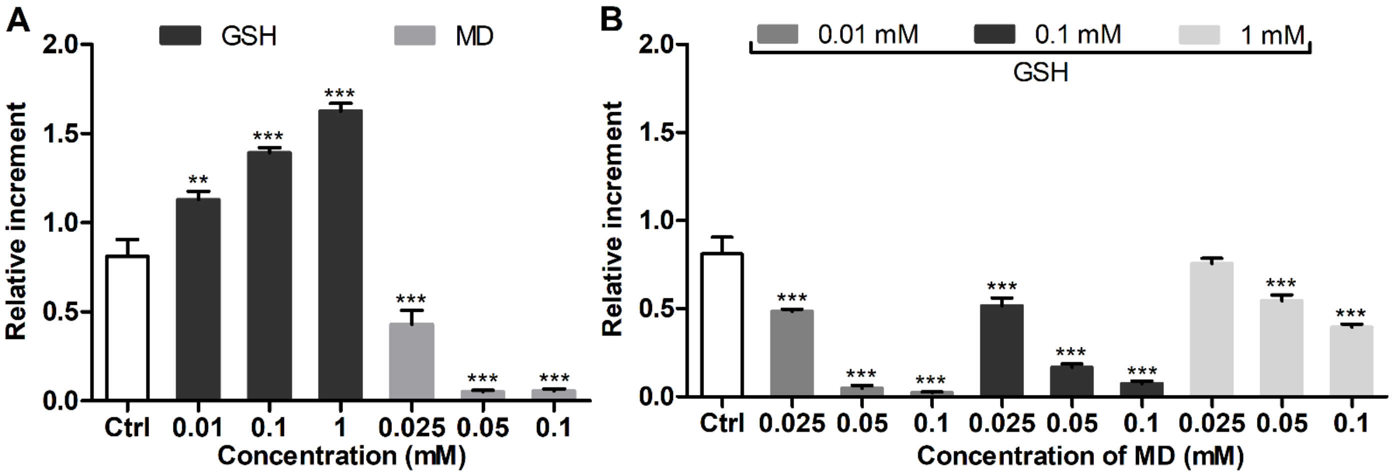

Figure S1. Effect of (**A**) supplemented GSH or menadione (MD) and (**B**) their mixture on MCB signal in 3T3 fibroblasts. The cells were treated with modulator(s) for 2 h and stained with MCB (in the presence of modulators). Mean ± SEM (n = 6, *$p < 0.05$, ***$p < 0.001$ vs. Ctrl) are shown; Ctrl is the signal of MCB alone.

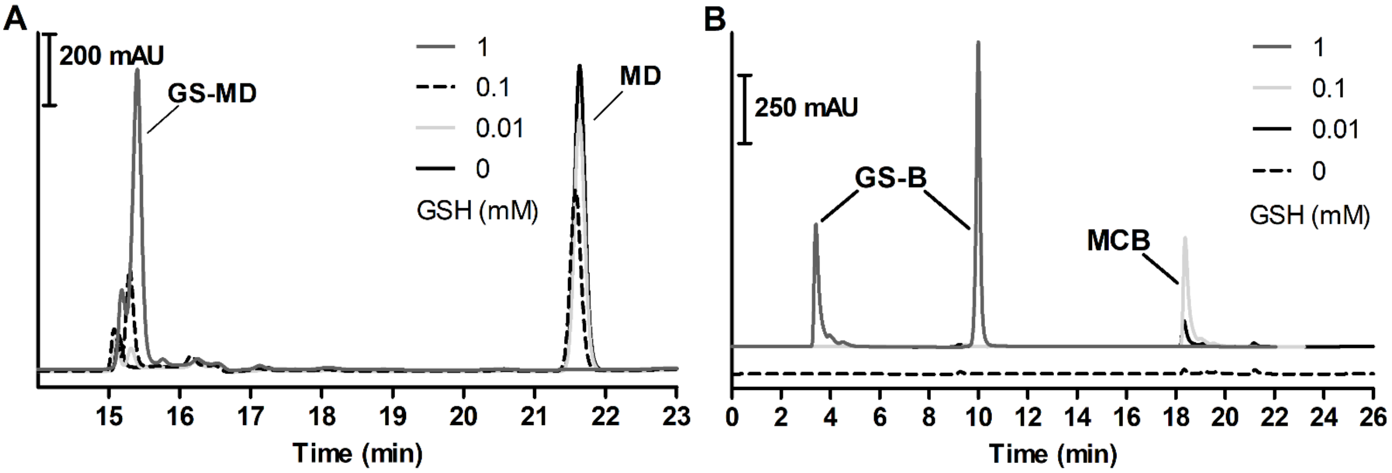

Figure S2. Reverse-phase HPLC chromatograms of (**A**) menadione (0.2 mM) and (**B**) MCB (0.01 mM) incubated with GSH (0.01–1 mM) in HBSS for 60 min. Water/acetonitrile mobile phase, flow rate 0.5 mL/min; (**A**) UV detection (λ = 220 nm), (**B**) fluorescence detection (λ_{em} = 482 nm). Adducts of GSH with menadione (GS-MD) and MCB (GS-B) were assigned.

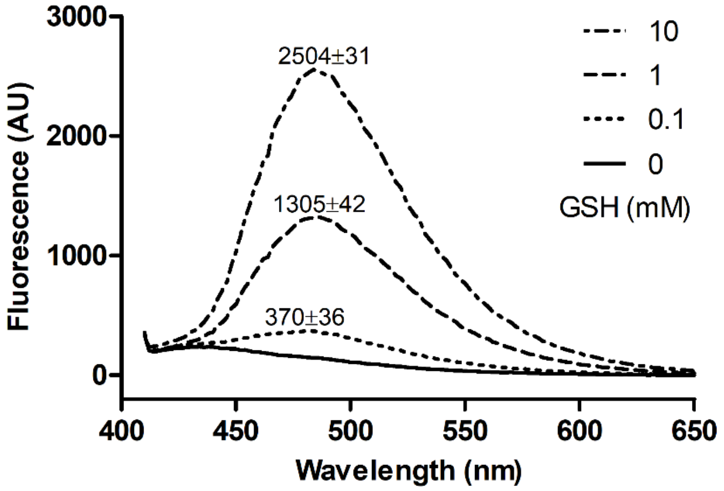

Figure S3. Fluorescence emission spectra of 5 μM MCB in HBSS in the presence of GSH. Mean fluorescence intensities are indicated for each GSH concentration (0.1, 1, 10 mM).

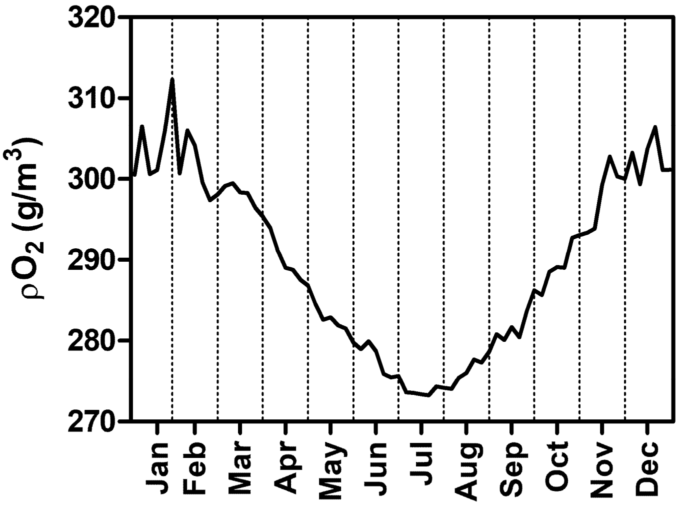

Figure S4. Annual dynamics of partial density of oxygen in Kazan with a typical humid continental climate (average data for 5 recent years in the city). The data were obtained from All-Russia Research Institute of Hydrometeorological Information per request of the authors.

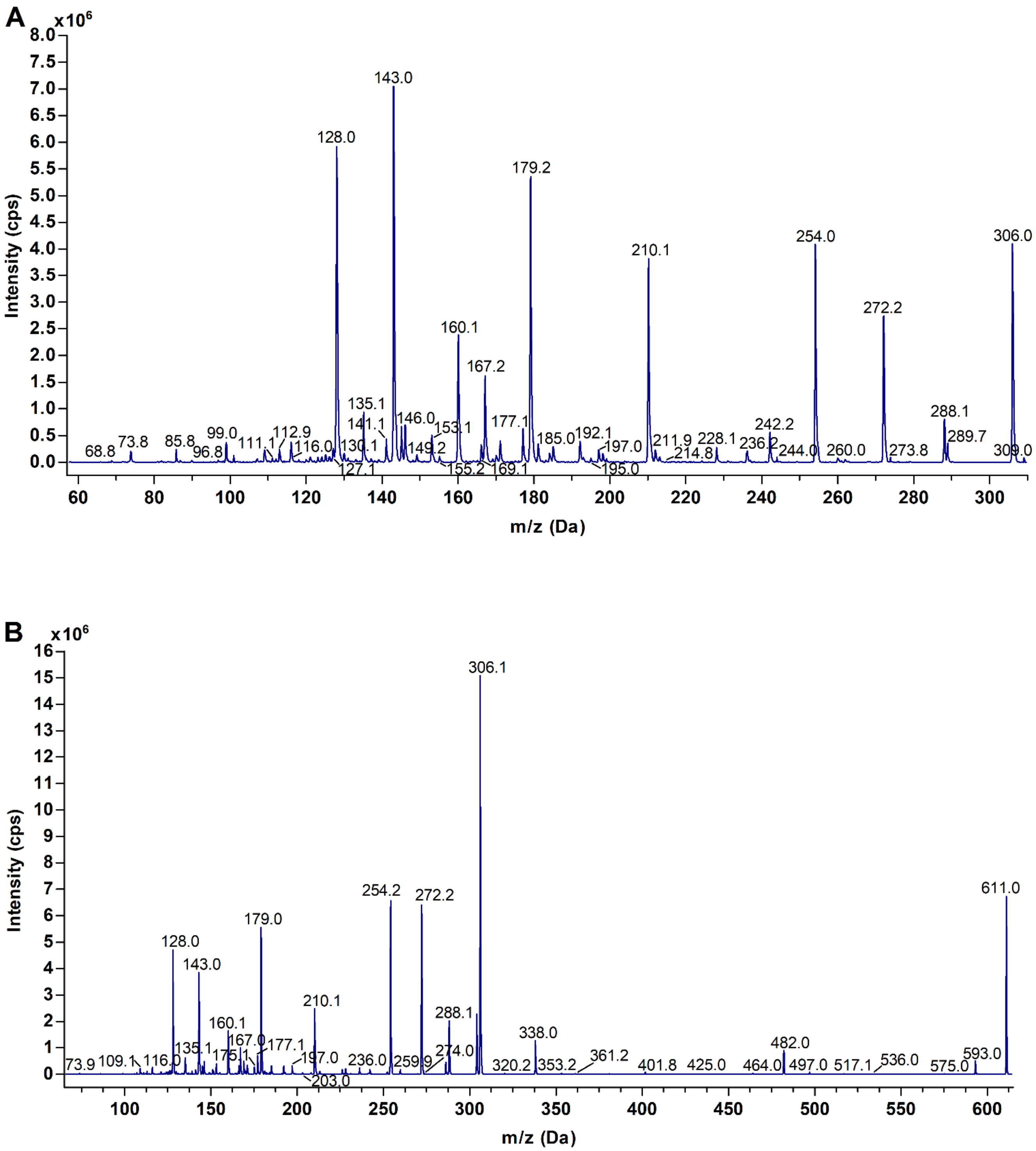

Figure S5. Negative ion spectra generated in flow injection analysis of (**A**) GSH and (**B**) GSSG.

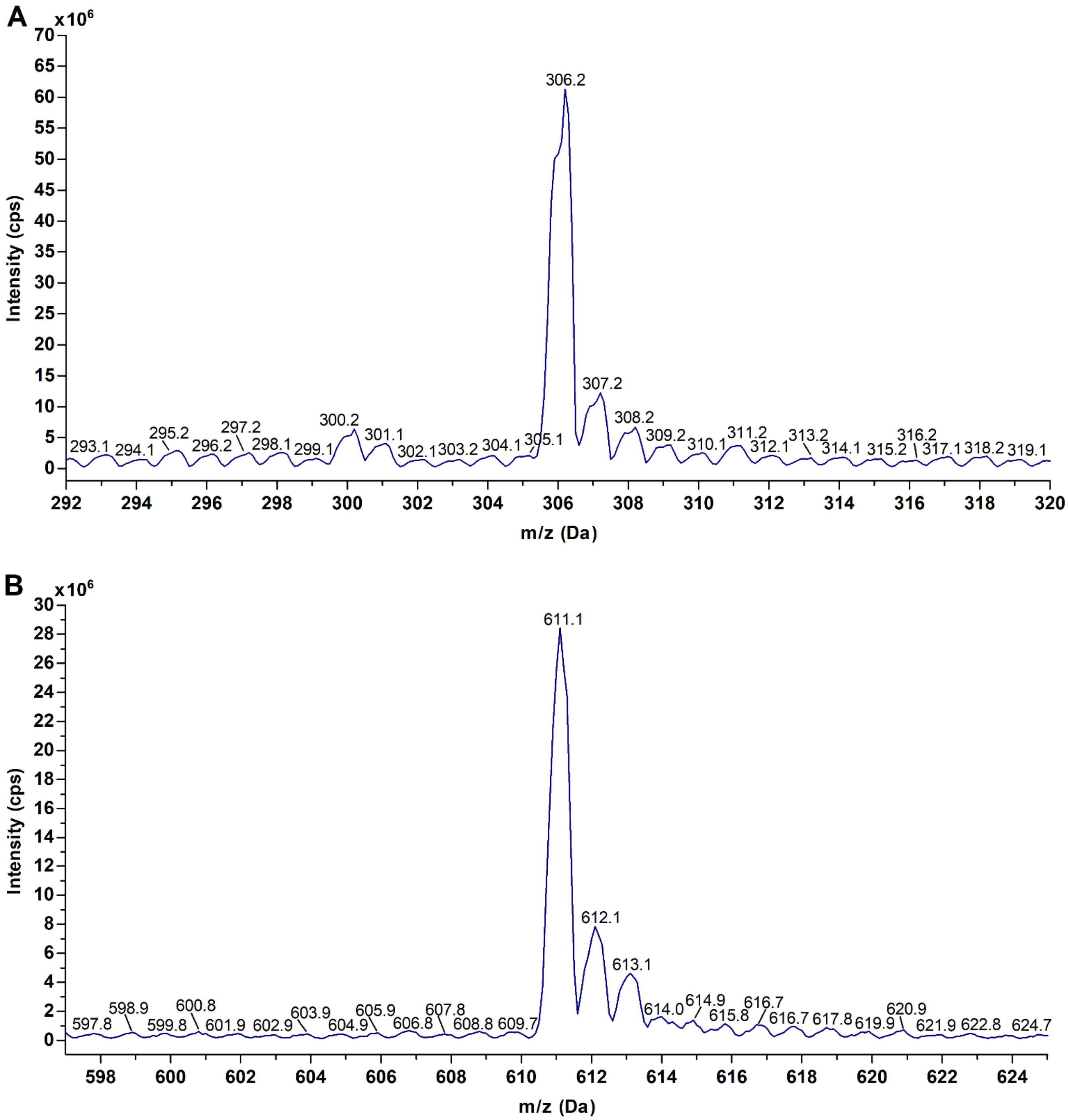

Figure S6. Product ion spectra obtained from (**A**) GSH and (**B**) GSSG parent ions.

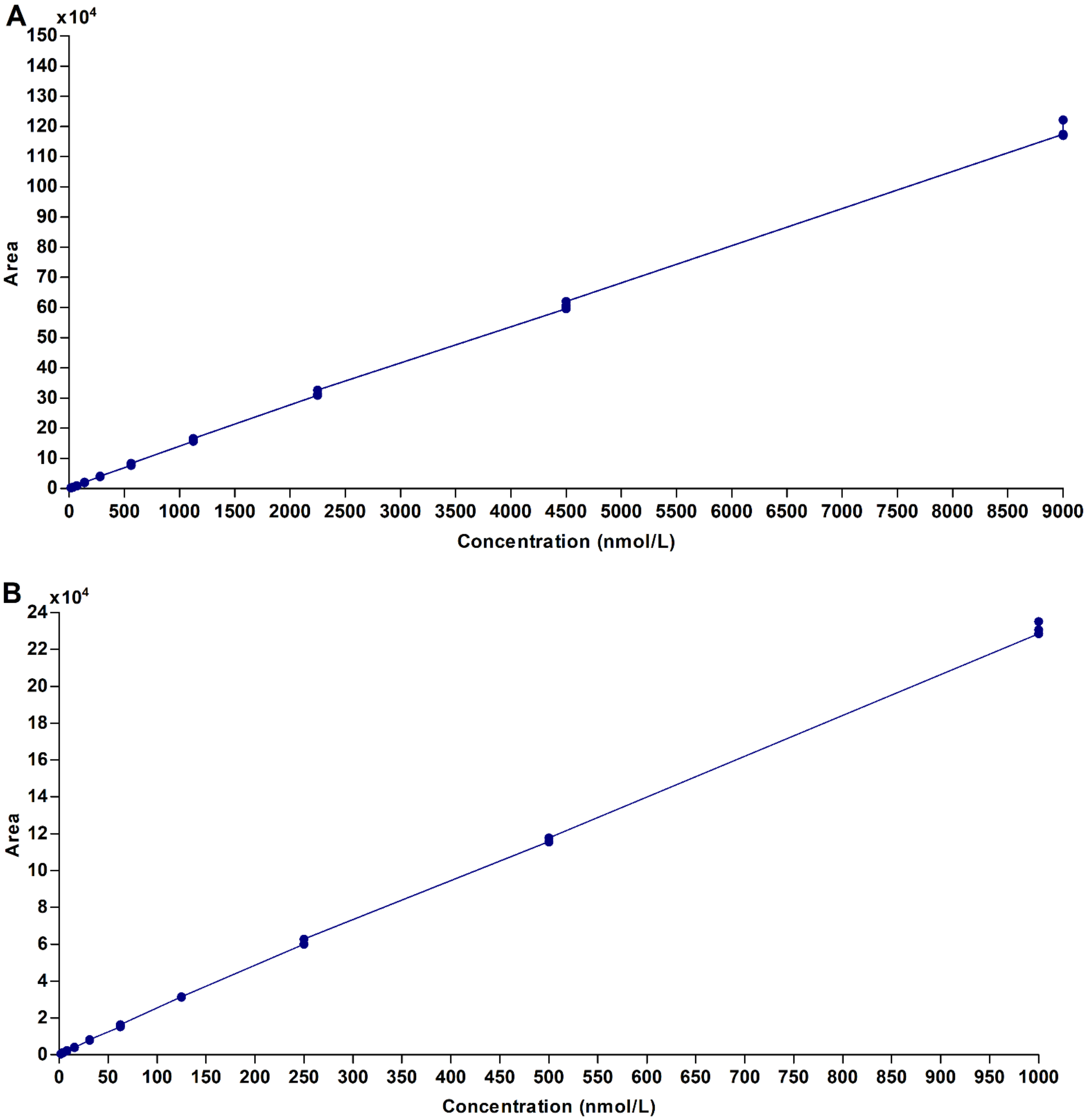

Figure S7. Calibration graphs for (**A**) GSH and (**B**) GSSG standards.
